# Sensitivity Analyses for Missing in Repeatedly Measured Outcome Data

**DOI:** 10.1002/sim.70282

**Published:** 2025-10-07

**Authors:** James F. Troendle, Aparajita Sur, Eric S. Leifer, Tiffany Powell‐Wiley

**Affiliations:** ^1^ Office of Biostatistics Research Division of Intramural Research of the National Heart, Lung, and Blood Institute, NIH/DHHS Bethesda Maryland USA; ^2^ Department of Biostatistics University of Minnesota Minneapolis Minnesota USA; ^3^ Social Determinants of Obesity and Cardiovascular Risk Laboratory Division of Intramural Research of the National Heart, Lung, and Blood Institute, NIH/DHHS Bethesda Maryland USA

**Keywords:** delta‐based controlled imputation, linear mixed model, multiple imputation

## Abstract

We discuss practical aspects of conducting sensitivity analyses for missing data with a repeatedly measured outcome. Our motivation is a SMART trial with a repeatedly measured outcome subject to missingness. We discuss and describe delta‐based controlled imputation approaches to conducting sensitivity analyses for such trials that typically use linear mixed models for their primary analysis. We find that delta‐based sensitivity analyses for trials with repeatedly measured outcome variables are enhanced by using MICE for the imputation. Further, including last‐observed‐before‐time covariates is critical for a repeatedly observed outcome. We also develop some novel metrics for judging the adequacy of sensitivity analyses.

**Trial Registration:** Tailoring Mobile Health Technology to Reduce Obesity and Improve Cardiovascular Health in Resource‐Limited Neighborhood Environments: NCT03288207.

## 1 Introduction

Missing data is prevalent in almost all clinical trials. Trials that rely on a repeatedly measured outcome will inevitably have missing outcome data. Many trials use a linear mixed model (LMM) of a continuous outcome assessed repeatedly post‐baseline for their primary analysis, where the effect of intervention at the time of interest is obtained through contrasts of the model parameters. Such models are preferable to models of only the outcome at the time of interest with regard to sensitivity to missing outcome data, but still rely on assumptions about the missing data. For this reason, sensitivity analyses assessing the impact of the type of missingness on trial conclusions are often recommended [1, 2]., including by the US Food and Drug Administration (FDA) [3].

Our motivation comes from the National Heart, Lung, and Blood Institute's (NHLBI) Step‐It‐Up (SIU) trial (NCT03288207). SIU has the potential for two randomized interventions to be given to each participant. The interventions are described in detail elsewhere [4, 5]. The target recruitment is for at least 130 participants (black overweight/obese women) who are randomized and complete follow‐up. Recruitment started in September 2021, with a total accrual (not all randomized) of 115 participants by June 2024. After a two‐week run‐in period, participants are randomized (1:1) to intervention A=0 (standard remote coaching) or A=1 (tailored‐to‐place coaching). Participants are followed for 12 weeks (stage 1), with weekly step counts being collected.

Average daily step counts over post‐baseline weeks 9–11 are used to categorize each participant as responder (≥10000 steps/day) or non‐responder (<10000 steps/day). However, if a participant has no measured step counts in weeks 9–11, they are classified as a non‐responder. After 12 weeks, non‐responders are re‐randomized (1:1) to intervention (tailored‐to‐place if A=0; tailored‐to‐place with increased messaging if A=1) or to intervention (standard remote coaching with face‐to‐face if A=0; tailored‐to‐place with face‐to‐face if A=1). Responders continue with the randomized intervention from the first stage (A). Participants are followed for an additional 12 weeks (24 weeks post‐baseline in total; stage 1 is weeks 1–12 and stage 2 is weeks 13–24), with weekly step counts being collected. Step count totals averaged over weeks 21–24 comprise the primary outcome. The focus of this work is on estimating the effect of the policy of intervention A on the primary outcome. Thus, our estimand is the average difference in steps/week over weeks 21–24 between participants randomized to A=1 versus A=0.

For this primary analysis, we ignore the effect of re‐randomization for several reasons. Firstly, inclusion of the effects of re‐randomization requires inclusion of many additional parameters in our models that would substantially increase the variance of our estimate of the effect of intervention A policy. Secondly, the question of intervention policy is of great practical importance. Thirdly, we do not anticipate the re‐randomized intervention to have a large effect (beyond that of intervention A) on missingness. If one did anticipate re‐randomization to have a large additional effect on missingness, one could add additional terms to the models in Section 2.2. Secondary analyses (not described here) will examine comparisons between embedded treatment regimes that naturally will include effects of the re‐randomized intervention assignments.

To describe the types of sensitivity analyses possible for such trials, we first recall the different types of missing data [6]. The missing data are called *missing completely at random* (MCAR) if missingness does not depend on the values of the data. A less restrictive assumption that the missing data are *missing at random* (MAR) holds if the probability of missingness does not depend on the unobserved values conditional on the observed values [6]. If MAR does not hold, the missing data are said to be *missing not at random* (MNAR).

It is well known [6] that an analysis of complete cases is valid under the restrictive MCAR assumption. For example, suppose in the SIU trial we made comparisons between the observed weeks 21–24 step counts for participants randomized to A=0 and participants randomized to A=1. This might be implemented via a two‐sample t‐test. The resulting comparison would be valid if (in addition to either the data being Gaussian or an asymptotic viewpoint was taken) the missing data were MCAR.

It is also known that analysis via a linear mixed model of the post‐baseline step count values is generally valid (in addition to the distributional assumptions of the LMM) under the broader MAR scenario. For example, suppose in the SIU trial we fit an LMM to all of the observed post‐baseline step count values and estimated a contrast representing the difference between the A=0 and A=1 arms for weeks 21–24. The comparison would be valid if the missing data were MAR. One way to view the MAR assumption when a model is used in analysis is that, conditional on the observed data in your model, the missing data are MCAR. Naturally, this means that the LMM analysis is preferable to the t‐test analysis with respect to the robustness of the conclusions to missing data. Moreover, the more outcome data that can be obtained and included in the LMM makes the conclusions less affected by MNAR deviations from MAR since the LMM conditions on all of the observed data. For this reason, inclusion of covariates that might predict missingness in the LMM is helpful in reducing the impact of missingness on the estimated treatment effect. However, the analyst rarely knows for certain that missing data are MCAR or MAR. Thus, it is imperative that sensitivity analyses are performed to judge the robustness (to missing data) of the results from an LMM analysis.

In this paper, we discuss and explore modeling approaches for trials with a repeatedly observed outcome. Further, we will describe and explore options for sensitivity analyses for MNAR departures from MAR missing. All of our ideas will be illustrated with examples of how this might be implemented in the SIU trial. We begin in Section 2 by describing modeling options for the SIU trial. Section 3 describes the data generation models used to simulate SIU‐like data. We explore methods of controlled multiple imputation in Section 4. Sections 5 & 6 describe and report the results of our investigations of sensitivity analyses by simulation. Finally, Section 7 gives discussion and recommendations for implementing sensitivity analyses in trials with repeatedly measured outcomes.

## 2 Modeling Approaches for Trials With a Repeatedly Measured Outcome

We describe two modeling approaches to analyze the repeated outcome data to determine the primary effect of the intervention A policy. For the SIU trial, the outcomes are the weekly step count averages. The first approach models only the outcome of interest (average of step counts in weeks 21–24). This method has the drawback that, since no intermediate outcome data are included in the model, it requires missingness to be MAR based on conditioning only on baseline covariates. We then describe a more robust (to missing outcome data) LMM for the SIU trial that models all of the outcome data. This latter model also requires missingness to be MAR, but based on conditioning on baseline covariates as well as all observed intermediate outcomes.

### 2.1 Linear Model Approach

A simple method to analyze the trial's primary outcome of the average of weeks 21–24 step counts would be to use a linear model. More specifically, let $Y_{i0}$ represent the average steps per day during the run‐in for participant i, while $Y_{i1},\ldots ,Y_{i24}$ represent the weekly post‐baseline average steps per day for participant i. Let $Y_{i}$ represent the average of $Y_{i21},Y_{i22},Y_{i23},Y_{i24}$, the trial's primary outcome. Let $A_{i}$ be the randomized value for the first stage randomization for participant i. Finally, let $AGE_{i}$ be the age in years of participant i at baseline. We will include $AGE_{i}$ in our models because SIU included it as a pre‐specified covariate. In general, all pre‐specified covariates would be included. Choice of covariates for inclusion is based on their having a prognostic effect on the outcome. Then the following simple linear model (LM) can be fit to the trial data from a total of n participants:

(1) $$\begin{array}{ll} Y_{i} & =\alpha +\beta_{age}AGE_{i}+\beta_{r}Y_{i0}+\beta_{A}A_{i}+\epsilon_{i} \end{array}$$

where $\epsilon_{i}$ is assumed to be an independent, normally distributed error. Let $\hat{\alpha },{\hat{\beta }}_{AGE},{\hat{\beta }}_{r},{\hat{\beta }}_{A}$ be the least squares fitted values of the parameters in Equation (1).

The effect of intervention A=1 policy is given from Equation (1) as $\beta_{A}$. The fitted value ${\hat{\beta }}_{A}$ is used as an estimator of the effect of the intervention A=1 policy. Testing is based on the standardized test statistic derived from ${\hat{\beta }}_{A}$, and relies on the asymptotically normal distribution of the fitted parameters.

### 2.2 Linear Mixed Model Approach

As has been mentioned, to obtain an unbiased estimate of the A intervention policy effect, the LM model (1) requires the missing data to be MCAR given the baseline covariates in the model. The linear mixed model (LMM) will relax that assumption.

Let t index post‐baseline time in weeks and let I() be the indicator function. We are now ready to give the mean model for the repeated weekly step count data:

(2) $$\begin{array}{ll} Y_{it} & =\beta_{age}\;AGE_{i}+\beta_{0}\;I(t=0)+\beta_{1}\;I(t>0)+\beta_{t1}\;t+\beta_{t2}t^{2}\\ & \;+\beta_{t3}\;t^{3}+\beta_{At}\;A_{i}\;I(t>0)+\epsilon_{it}\;t=0,\ldots ,24 \end{array}$$

where $\epsilon_{it}$ is assumed to be a normally distributed error, with independence between participants and an autoregressive first‐order AR(1) correlation structure within a participant. Note that model (2) makes no assumptions about intervention effects over time, while properly constraining the outcomes to be similar between intervention arms during the run‐in at t=0. Furthermore, model (2) accounts for the participants' age at baseline. Finally, model (2) allows a cubic time trend over the intervention period, plus a term for the run‐in at t=0.

Next, we define the random effects that will model the correlation over time of weekly step counts within a participant. Let $b_{i}$, $b_{1i}$ be two random effects that apply respectively to each observation for participant i. The first random effect, $b_{i}$, is an intercept. The second random effect, $b_{1i}$, is a slope; it is multiplied by the time t. These random effects are assumed to be normally distributed and independent for different i. The LMM can now be written as

(3) $$\begin{array}{ll} Y_{it} & =\beta_{age}\;AGE_{i}+\beta_{0}\;I(t=0)+\beta_{1}\;I(t>0)+\beta_{t}\;t\\ & \;+\beta_{t2}t^{2}+\beta_{t3}\;t^{3}+\beta_{At}\;A_{i}\;I(t>0)+b_{i}\\ & \;+b_{1i}\;t+\epsilon_{it}\;t=0,\ldots ,24 \end{array}$$

We will use the same convention as was used in Section 2.1 that ∧ above a parameter refers to the fitted value from Equation (3). Let ${\hat{\sigma }}_{res}$ be the estimated residual standard deviation from the LMM model (3).

Recall that the primary intervention policy effect is based on step counts averaged over weeks t=21 to t=24. The effect of intervention A=1 policy is given from LMM model (3) as

(4) $$\begin{array}{ll} \theta_{A} & =\frac{1}{4}(\beta_{A21}+\beta_{A22}+\beta_{A23}+\beta_{A24}) \end{array}$$

Plugging in fitted values gives

(5) $$\begin{array}{ll} {\hat{\theta }}_{A} & =\frac{1}{4}({\hat{\beta }}_{A21}+{\hat{\beta }}_{A22}+{\hat{\beta }}_{A23}+{\hat{\beta }}_{A24}) \end{array}$$

as an estimator of the effect of the intervention A=1 policy. Testing is based on the standard model estimated standard deviation (for this contrast).

## 3 Simulation of SIU‐like Data

To understand the properties of various primary analysis and sensitivity analysis options, we simulate data similar to that expected in the SIU trial. First, intervention assignment $A_{i}$ was generated as an independent Bernoulli(0.5), and age at randomization was generated as U(20,40). An LMM was used to generate SIU‐like step count for the run‐in (weeks −1 and 0) and weeks 1–24, with the model given by

(6) $$\begin{array}{ll} Y_{it} & =\alpha^{*}+\beta_{age}^{*}\;AGE_{it}+\beta_{0}^{*}\;I(t<1)+\beta_{A}^{*}\;A_{i}+c0_{i}+c1_{i}t+\eta_{it}\\ & t=-1,\ldots ,24 \end{array}$$

where $AGE_{it}$ is the age in years of participant i at the end of week t, $c0_{i}$, $c1_{i}$ and $\eta_{it}$ will be discussed below, and the symbols super‐scripted with ∗ are input parameters.

While this generation model is overly simplified in that it uses constant intervention effects across time, we believe it is sufficient for the generation of SIU‐like data to explore statistical properties. Note that the LMM model (3) is our proposed analysis model, which appropriately does allow arbitrary intervention effects across times.

Correlation within a participant enters (6) via the $c0_{i},\;c1_{i}$ and $\eta_{it}$ terms. The $c0_{i},\;c1_{i}$ terms represent an independent Gaussian random intercept and slope for participant i, with zero means and standard deviations $\sigma_{c0},\;\sigma_{c1}$. The $\eta_{it}$ (independent for different i and independent of $c0_{i}$ and $c1_{i}$) within each i are 26 dimensional multivariate Gaussian error with zero mean and spatial correlation $\rho (t_{1},t_{2})=\exp(-|t_{1}-t_{2}|/52)$ and equal standard deviation $\sigma_{e}$.

Data generated from Equation (6) was subjected to missingness by generating indicators of missing, $R_{it}$, according to logistic models. Details of the models are given in section 8.1 of the Supporting Information. The models allow missingness to depend (along with an intercept $\alpha_{m}$) on $A_{i}$ (through coefficient $\beta_{ma}$), the number of steps during the run‐in (through coefficient $\beta_{m0}$), time t (through coefficient $\beta_{mt}$), $Y_{il_{t}}$ (the last previously observed value for participant i at time t; through coefficient $\beta_{ml}$), the interaction of $Y_{il_{t}}$ with $A_{i}$ (through coefficient $\beta_{mla}$), $Y_{it}$ (through coefficient $\beta_{my}$), the interaction of $Y_{it}$ with $A_{i}$ (through coefficient $\beta_{mya}$), and after potential re‐randomization (weeks 13–24) missingness could also depend on the outcome $(Y_{i21}+Y_{i22}+Y_{i23}+Y_{i24})/4$ (through coefficient $\beta_{mr}$). The models allow for a large range of missingness that includes MCAR and many types of MAR or MNAR. Throughout this paper and in the tables, we use MAR and MNAR to refer to missing with respect to the LMM model (3).

### 3.1 Results

Table 1 shows results of bias and type I error for testing via the LM model (1) or via the LMM model (3). The data were generated from the LMM (6). In Table 1, the type I error from either the LM model (1) or the LMM model (3) using the standard model estimated variance is reasonably controlled (and the estimates are essentially unbiased, that is, if there is any bias it is quite small compared to the average of 8500 steps/day in Equation (6)) when the data are generated from Equation (6) with MCAR missing data. In contrast, the fourth row shows that with MAR missing data, estimation and testing via the LM model (1) can exhibit significant bias and excess type I error. The last row demonstrates that with MNAR missing data, estimation and testing via either the LM model (1) or the LMM model (3) can exhibit significant bias and excess type I error.

**TABLE 1:** Estimated bias ± 2SE of step counts (% type I error)^a^ (%)^b^ of treatment policy effect for simulated SIU‐like trials ($n=130,\alpha^{*}=10\;000,\beta_{age}^{*}=-50,\beta_{0}^{*}=-4000$, treatment policy effect $\beta_{A}^{*}=0,\sigma_{c0}=\sigma_{c1}=50,\sigma_{e}=2000$). Missingness for MAR and MNAR scenarios according to models (S1) and (S2) from section 8.1 of the Supporting Information and with zero parameters except those listed in the table and $\alpha_{m}=-1.5,\beta_{ma}=1,\beta_{mt}=1/12$. Missingness for MCAR has $\beta_{m0}=\beta_{ma}=\beta_{mt}=0$. Average step count = 8500 steps/day.

Missing	$\beta_{ml}$	$\beta_{mla}$	$\beta_{m0}$	$\beta_{my}$	$\beta_{mya}$	$p_{\text{miss}}$ ^c^	Model	
							LM (1)	LMM (3)
MCAR	0	0	0	0	0	0.00	−5±6.7 (5.32)	−5± 6.6 (5.49)
MCAR	0	0	0	0	0	0.20	−5± 6.7 (5.42)	−6± 6.6 (5.43)
MAR	0	0	0	0	0	0.21	−5± 6.8 (5.32)	−6± 6.7 (5.33)
MAR	0.001	0.001	0	0	0	0.10	−273± 6.7 (13.25)	−1± 6.8 (5.55)
MNAR	0.001	0.001	−0.0005	−0.002	−0.002	0.20	391 ± 6.9 (19.86)	285 ± 6.4 (14.38)

^a^ Estimated over 10 000 replicated trials; target rejection rate is 5% under null.

^b^ Standard error of type I error estimates over the 10 000 replications is 0.22%.

^c^ $p_{\text{miss}}$ is average overall proportion of outcome values missing.

## 4 Sensitivity Analysis of SIU

Here, we describe options for sensitivity analysis of missing outcome data for a trial like SIU. Before we describe the methods, we need to define some notation. Let ${\text{Y}}_{i}=\{Y_{i0},\ldots ,Y_{i24}\}$ be the vector of outcome data for participant i and ${\text{X}}_{i}$ be the vector of covariates (which in general can include treatment assignments and indicators of response to treatment). Let $R_{it}$ be the observed indicator for $Y_{it}$, while ${\text{R}}_{i}$ is the vector of such indicators for participant i. Let Ω be a vector of parameters, and $f(Y_{i}|X_{i},\Omega )$ be the distribution of the outcome data. Let Φ be a vector of parameters defining the missing data mechanism through $f({\text{R}}_{i}|{\text{Y}}_{i},{\text{X}}_{i},\Phi )$.

We follow Moreno‐Betancur and Chavance and Cro et al. [1, 2], and adopt a *pattern‐mixture model* approach to specification of the joint distribution of the observed data and missingness process. Let ${\text{Y}}_{i}$ be partitioned into $\left\{{\text{Y}}_{iO},{\text{Y}}_{iM}\right\}$ where ${\text{Y}}_{iO}$ is the observed part and ${\text{Y}}_{iM}$ is un‐observed. Thus, we need to specify the terms on the right‐hand side of

(7) $$\begin{array}{ll} & f({\text{Y}}_{i}|{\text{X}}_{i},\Omega )f({\text{R}}_{i}|{\text{X}}_{i},\Phi )\\ & \;=f({\text{Y}}_{iM}|{\text{Y}}_{iO},{\text{R}}_{i},{\text{X}}_{i},\Omega )f({\text{Y}}_{iO}|{\text{R}}_{i},{\text{X}}_{i},\Omega )f({\text{R}}_{i}|{\text{X}}_{i},\Phi ) \end{array}$$

The second term on the right‐hand side of (7) is our analysis LMM (3), and the third is implicitly defined empirically by our application of imputation on the missing values. Thus, in practice, a pattern mixture approach is about specification of $f({\text{Y}}_{iM}|{\text{Y}}_{iO},{\text{R}}_{i},{\text{X}}_{i},\Omega )$. Controlled imputation methods make this specification by starting with the distribution suggested by an imputation model (or models), and then systematically modifying them for each pattern of missing. Our imputation models for the SIU trial contain the variables in Equation (3) along with the last observed Y before the current time t. Although for the SIU trial we end up using an imputation method that uses predictive mean matching (see section 8.2 of the Supporting Information for details) as opposed to draws directly from a predictive distribution from a single imputation model, for simplicity of language we describe the general multiple imputation and sensitivity analyses as though a single imputation model was used. In general, one could use the same model for analysis and for imputation, but often the imputation model will contain additional covariates that help predict missingness or are needed for the particular pattern mixture being used. There are two general classes of controlled imputation approaches, *delta‐based* and *reference‐based*, but we focus here on *delta‐based* [2].

An alternative to *pattern‐mixture models* is called using a *selection model* approach, which requires specification of the left‐hand side of (7). Although there are sensitivity approaches using a *selection model* [7], it may be easier to conduct sensitivity analyses according to a *pattern‐mixture model* approach because of the natural starting point that controlled imputation methods use under a MAR missing assumption in estimation of $f({\text{Y}}_{iM}|{\text{Y}}_{iO},{\text{R}}_{i},{\text{X}}_{i},\Omega )$ from an imputation model. Note that an imputation model should include in ${\text{X}}_{i}$ the variables in the analysis LMM for $f({\text{Y}}_{iO}|{\text{R}}_{i},{\text{X}}_{i},\Omega )$, but could also include auxiliary covariates. Usually, auxiliary covariates are included in ${\text{X}}_{i}$ because they could help predict missingness, although there are sometimes special circumstances where variables not predictive of missingness are included in the imputation model, like when one wants the pattern mixture to depend on those variables. We will have more about the specific variables included in ${\text{X}}_{i}$ used in the imputation models compared in this paper in Section 5.1.

### 4.1 Multiple Imputation

The idea of multiple imputation has a long history dating to the 1970s [8]. There are many good references for general approaches to multiple imputation [6, 9, 10]. Controlled imputation in particular [1, 2, 11], and multiple imputation through the use of chained equations [12, 13] has gained popularity over the past two decades.

The basic steps of a multiple imputation analysis are briefly summarized here. Let M be a large integer. You will generate M complete datasets, so for a real application, M should be large enough that the Monte‐Carlo error on your results is small: Perhaps 100 or larger. For simulations, M can be much smaller as the conclusions will be based on averaging over many simulation replications and thus much less influenced by the Monte‐Carlo error of a small M. The following algorithm assumes you have an analysis model and an imputation model fit on the observed data.

**General MI Algorithm with imputation and analysis models**

**Step 0:** For m=1,…,M repeat Steps 1–3.

**Step 1:** Replace each missing value in the observed dataset by a random draw from the predictive distribution of the fitted imputation model, obtaining the $m^{th}$ complete dataset.

**Step 2:** Fit the analysis model on the $m^{th}$ complete dataset.

**Step 3:** Obtain the $m^{th}$ estimate of intervention policy effect, ${\hat{\theta }}_{A}^{m}$, and its model estimated variance.

**Step 4:** Average the ${\hat{\theta }}_{A}^{m}$ to get the multiple imputation estimate, ${\hat{\theta }}_{A}^{MI}$.

**Step 5:** Use Rubin's rules [6] to get $\hat{Var}({\hat{\theta }}_{A}^{MI})$, the estimate of the total variance of ${\hat{\theta }}_{A}^{MI}$, including the between and within imputation variances.

**Step 6:** Conduct hypothesis testing of $H_{0}:\theta_{A}\leq 0$ based on asymptotic normality of the standardized ${\hat{\theta }}_{A}^{MI}$. For example, a one‐sided test rejects if ${\hat{\theta }}_{A}^{MI}/\sqrt{\hat{Var}({\hat{\theta }}_{A}^{MI})}>1.96$.

### 4.2 Delta‐based Controlled Imputation Approaches

*Delta‐based* controlled imputation methods specify a $\delta_{\max}$ for each pattern of missing. The specification of our pattern mixture model $f({\text{Y}}_{iM}|{\text{Y}}_{iO},{\text{R}}_{i},{\text{X}}_{i},\Omega )$ is often obtained by using the predicted distribution of the fitted imputation model evaluated at ${\text{Y}}_{iM}$, and modifying it by adding a fraction (δ) of $\delta_{\max}$ (thus $f({\text{Y}}_{iM}|{\text{Y}}_{iO},{\text{R}}_{i},{\text{X}}_{i},\Omega )$ accounts for $\delta_{\max}$). For example, in the case where we treat all missing patterns the same, each missing value is imputed from the predictive distribution implied by the imputation model $+\;\delta \cdot \delta_{\max}$.

We describe one version of *delta‐based* controlled imputation sensitivity analyses, which we call *SD sensitivity*. This *SD‐sensitivity* version specifies a single fixed $\delta_{\max}$. Formally, we set $\delta_{\max}$ to be a fixed multiple, λ, of the residual sample standard deviation from the observed data fit of the LMM model (3). This *SD‐sensitivity* version treats all missing patterns the same. Thus, when imputing for a missing value, we use a value from the predictive distribution of the imputation model $+\delta \cdot \delta_{\max}$. This means that we assume the missing values on average differ from what the imputation model would predict by no more than $\delta_{\max}=\lambda \cdot \hat{SD}$, where $\hat{SD}$ is the residual sample standard deviation from the observed data fit of LMM model (3). We use λ=2 as a natural choice. This choice is arbitrary, but since λ=2 yields a $\delta_{\max}$ that is two standard deviations, imputed values corresponding to $\delta_{\max}$ will deviate from the imputation model predicted value by two standard deviations. Other pattern mixture approaches could allow $\delta_{\max}$ to be a function of time or other factors, allowing for a more complex investigation where the type of missingness defines the distribution of the unobserved values.

A sensitivity analysis uses multiple imputation S+1 times (once for each δ value including 0), where S is an even positive integer chosen so that the grids {δ=−2r/S:r=S/2,…,1} and {δ=2r/S:r=1,…,S/2} have the desired level of granularity. One follows the general MI algorithm given above S+1 times, with each imputation modified by adding $\delta \cdot \delta_{max}$ to generate S+1 estimated treatment effects ${\hat{\theta }}_{A1},\ldots ,{\hat{\theta }}_{AS+1}$ with the associated hypothesis tests.

### 4.3 Sensitivity Analysis Summary Metrics

In evaluating a sensitivity analysis via simulation, we propose tracking some new parameters described here. These parameters take a single value for each observed dataset (single replication of a simulation experiment) based on the observed data analysis of (3) along with the various sensitivity MI analyses (with various delta values) described above. The first is based on the concept of a *sensitivity interval of rejection* (SIR). We define an SIR as a symmetric interval of δ about 0 with positive width, on which the null is rejected for each corresponding sensitivity analysis, as well as for the analysis LMM (3) fit on the observed data (the one‐sided tests must agree). We define our measures to be symmetric intervals of delta because we do not usually want to assume delta to be either positive or negative. Consider an example with S=8: The sensitivity deltas are thus {−1,−3/4,−1/2,−1/4,0,1/4,1/2,3/4,1}. Suppose the LMM model (3) fit on the observed data rejects the null for a positive treatment policy effect. Suppose the set of deltas that also reject the null for positive treatment policy effect is {−1/2,−1/4,0,1/4}. Then we say there is an SIR:[−1/4,1/4] with half‐width 1/4. If there is no SIR, we define the half‐width to be 0 so that we can report the average half‐width across replications of simulated datasets, which may or may not have SIR.

Next, we define a *sensitivity interval of concordance* (SIC) as a symmetric interval of δ about 0, on which the one‐sided tests for each corresponding sensitivity analysis agree with the analysis of the LMM model (3) fit on the observed data. If either no deltas agree or if only δ=0 agrees, then we define the SIC to be [0,0] with half‐width 0. Thus, in the example above, we have SIC=SIR=[−1/4,1/4]. Note that these definitions are dependent on the value of S, that is, the increment for δ chosen for your sensitivity analysis. The parameters SIR and SIC are proposed as useful metrics to track the ability of sensitivity analyses to handle different types and amounts of missingness. To make these parameters meaningful, one should use an S of at least 8 (as we will in our simulations in Section 6), which implies an increment for δ of 1/4.

## 5 Multiple Imputation Implementation

We investigated three methods of multiple imputation. The first is described in section 8.2 of the Supporting Information and uses the R [14] package *mice* [12] with predictive mean matching (we denote this method as MICE). The MICE imputation method in our case uses predictive mean matching from a linear model fit to a single outcome variable to impute values that are sequentially used in further imputation of each outcome with missing values. We describe in some detail the MICE method in section 8.2 of the Supporting Information as it is included in our sensitivity simulation in Section 6. A second method is specifically designed for linear mixed models and described in Section 2.2 of Moreno‐Betancur and Chavance [1] (we denote this method as MB). The MB imputation method uses draws from an imputation model that is the same as the analysis model, with added variability to account for model uncertainty. A third method uses the R package *missForest* [15] (we denote this method as MF). The MF imputation method grows random forests that implicitly can account for interactions, making it very flexible.

### 5.1 Comparison of Imputation Methods

We used simulation to compare the three imputation methods on SIU‐like data (6) with missing data models (S1‐S2) from section 8.1 of the Supporting Information. The variables included in ${\text{X}}_{i}$ for the imputation model $f({\text{Y}}_{iM}|{\text{Y}}_{iO},{\text{R}}_{i},{\text{X}}_{i},\Omega )$ used by the MICE method (described in section 8.1 of the Supporting Information) include the last observed $Y_{it}$ before the current time t ($Y_{il_{t}}$) along with baseline covariates (AGE and $Y_{0}$) and each of the other $Y_{it}$ for t=1,…,24 for each missing $Y_{it}$, although the final imputation used predictive mean matching. The MF method also used these same covariates in its random forest imputation model. The MB method used the analysis LMM model (3) as its imputation model.

The addition of the last observed Y before the current time seems potentially important for longitudinal imputation models. That is because it might be thought that a participant doing particularly poorly (or particularly well) would be more or less likely to miss future measurements. One way to capture such information is through the last observed outcome.

Table 2 shows results of bias in the estimated treatment A=1 policy effect via the LMM model (3) under a null simulated data generation when imputing according to the three methods. Since this comparison only considered bias, we used M=1 for computational expediency. Estimation is averaged over imputations and then over replications, thus using M=1 with 10 000 replications is sufficient for accurate bias estimation. With data missing according to either MCAR or MAR, one hopes the estimate without imputing is unbiased. The table shows that this appears to be the case. When imputations are used, however, none of the methods are entirely unbiased for all missing scenarios. This might reflect that the MICE and MF methods do not impute according to the data‐generating model. We note, however, that the method MB, which does attempt to impute from an LMM (with random parameter draws to allow for uncertainty), which should contain the data‐generating model, actually has the highest bias. Simulations with n=260 (reported in section 8.3 of the Supporting Information) support the notion that small sample bias in the imputation models is responsible for much if not all of the bias in the MICE method. Also note that the MF method would presumably do relatively better if the missing depended on unknown interactions of the parameters included in the analysis LMM model (3). It seems that multiple imputing for LMMs is still an area that needs further research. The least biased methods across the scenarios considered here seem to be the MICE and MF methods. We chose the MICE method to be used in our sensitivity simulations in the next section because of its conceptual simplicity. This choice in no way means we believe that MICE is statistically better than MF.

**TABLE 2:** Estimated bias ± 2SE^a^ for treatment policy effect from LMM model (3) with various imputation methods applied to simulated SIU‐like trials ($n=130,\alpha^{*}=10\;000,\beta_{\text{age}}^{*}=-50,\beta_{0}^{*}=-4000,$ treatment policy effect $\beta_{A}^{*}=0,\sigma_{c0}=\sigma_{c1}=50,\sigma_{e}=2000,M=1$). Missingness for MAR scenarios according to models (S1) and (S2) from section 8.1 of the Supporting Information and with zero parameters except those listed in the table and $\beta_{ma}=1,\beta_{mt}=1/12$.

Missing	$\alpha_{m}$	$\beta_{ml}$	$\beta_{mla}$	$p_{\text{miss}}$ ^b^	No imp.	Imputation Method		
						MICE ^c^	MF ^d^	MB^e^
MCAR	0	0	0	0.20	−6± 6.6	−6± 6.6	4± 6.5	−4± 7.2
MCAR	0	0	0	0.40	−5± 6.7	−5± 6.5	14± 6.3	−8± 8.2
MAR	−1.5	0	0	0.21	−6± 6.7	−5± 6.5	−2± 6.3	−9± 8.2
MAR	−0.5	0	0	0.40	−4± 6.8	−2± 6.5	3± 5.9	−4± 9.3
MAR	−1.5	0.001	0	0.15	−5± 6.7	−14± 6.2	−5± 6.1	−95± 7.5
MAR	−1.5	0.001	0.001	0.15	−1± 6.8	−24± 6.2	−4± 6.2	−218± 7.7

^a^ Estimated over 10 000 replicated trials using (5).

^b^ $p_{\text{miss}}$ is average overall proportion of outcome values missing.

^c^ Imputation from *mice* R package with predictive mean matching described in section 8.2 of the Supporting Information.

^d^ Imputation from *missForest* R package.

^e^ Imputation from the method described in [1].

## 6 Sensitivity Simulations

We used simulation to investigate the properties of the proposed *Delta‐based* sensitivity analyses (Section 4.2) on SIU‐like data (6) with missing data from models (S1‐S2) of section 8.1 of the Supporting Information and using an MI strategy detailed in section 8.2 of the Supporting Information. The sensitivity analyses used S=8, which implies an increment for δ of 1/4 and an *SD‐sensitivity* version with λ=2. Recall that $\delta_{\max}$ (which determines the largest departure from the model predicted value for missing values at time t) is set to $\lambda \cdot \hat{SD}$, where $\hat{SD}$ is the residual sample standard deviation from our fit of (3).

Table 3 shows the results of bias and type I error for testing via model (3) under a null simulated data generation. The results are presented in the tables as bias (% type I error, % testing different from no imputation), so that in the first row of Table 3 the no imputation method has average bias of −6 steps/day with type I error of 5.4% (the − means that testing agreement with no imputation is automatic and so not applicable). The method using multiple imputation with δ=0 also has an average bias of −6 steps/day and type I error of 5.4%, with 0.6% testing disagreement with the method with no imputation. Thus, for 99.4% of the replications, these two methods agreed on the hypothesis test of treatment policy effect. The rows are indexed by k corresponding to eight missingness scenarios (missingness models are described in section 8.1 of the Supporting Information).

**TABLE 3:** Estimated bias^a^ (% type I error^b^, % different from no imputation^c^) for treatment policy effect from LMM model (3) with *SD‐sensitivity* version delta‐based sensitivity parameter δ applied to simulated SIU‐like trials ($n=130,\alpha^{*}=10\;000,\beta_{age}^{*}=-50,\beta_{0}^{*}=-4000,$ treatment policy effect $\beta_{A}^{*}=0,\sigma_{c0}=\sigma_{c1}=50,\sigma_{e}=2000,M=10$). Missingness scenarios indexed by k and described in text.

Missing		^d^	^e^	^f^	^g^		δ		
Type	k	$p_{\text{miss}}$	$p_{\text{SIR}}$	$len_{\text{SIR}}$	$len_{\text{SIC}}$	No imp.	−1	0	1
MCAR	1	0.20	0.039	0.025	0.952	−6 (5.4, —)	−6(3.9,4.1)	−6(5.4,0.6)	−8(4.1,4.0)
MCAR	2	0.40	0.034	0.021	0.947	−5 (5.4, —)	−5(4.1,4.5)	−5(4.8,1.0)	−7(4.1,4.7)
MAR	3	0.21	0.010	0.003	0.429	−6 (5.3, —)	−838(59.6,59.4)	−5(5.2,0.9)	825 (58.6, 58.8)
MAR	4	0.40	0.006	0.002	0.433	−4 (5.4, —)	−849(60.6,60.3)	−4(4.2,2.0)	838 (59.7, 59.9)
MAR	5	0.15	0.021	0.009	0.748	−5 (5.5, —)	−394(34.9,34.7)	−14(5.4,1.8)	374 (12.1, 12.1)
MAR	6	0.15	0.023	0.010	0.781	−1 (5.6, —)	−385(30.1,30.0)	−24(5.1,1.7)	341 (10.0, 9.8)
MNAR	7	0.08	0.031	0.016	0.885	17 (5.5, —)	150 (8.7, 9.5)	−19(5.3,1.8)	−190(9.1,8.5)
MNAR	8	0.20	0.043	0.025	0.822	285 (14.4, —)	−16(4.9,14.6)	113 (7.1, 8.7)	266 (17.5, 15.5)

^a^ Estimated over 10 000 replicated trials using (5); target rejection rate is 5% under null.

^b^ Standard error of type I error estimates over the 10 000 replications is 0.22%.

^c^ % of replications where the hypothesis test is different from the test without imputations.

^d^ $p_{\text{miss}}$ is average overall proportion of outcome values missing.

^e^ $p_{\text{SIR}}$ is rate of a sensitivity interval rejection.

^f^ $len_{\text{SIR}}$ is average half‐length of sensitivity interval of rejection.

^g^ $len_{\text{SIC}}$ is average half‐length of sensitivity interval of concordance.

The first two scenarios have purely MCAR missing. Scenario 3 has MAR missing with a logit intercept of $\alpha_{m}=-1.5$, and effect of time: $\beta_{mt}=1/12$. Scenario 4 (MAR) differs from scenario 3 only by having a larger logit intercept of $\alpha_{m}=-0.5$ so that there is more missing data. Scenario 5 (MAR) differs from scenario 3 only by the inclusion of an effect of the last observed value before the current time: $\beta_{ml}=0.001$. Scenario 6 (MAR) differs from scenario 5 only by the inclusion of an interaction effect of the last observed value before the current time and treatment: $\beta_{mla}=0.001$. Scenario 7 (MNAR) differs from scenario 3 by the inclusion of an effect of the number of steps during the run‐in of $\beta_{m0}=-0.0005$, and an effect of the number of steps in weeks 21–24: $\beta_{mr}=-0.002$. Scenario 8 (MNAR) differs from scenario 6 by inclusion of an effect of $Y_{it}$, $\beta_{my}=-0.002$, as well as an interaction effect of $Y_{it}$ with $A_{i}$: $\beta_{mya}=-0.002$, and an effect of the number of steps during the run‐in of $\beta_{m0}=-0.0005$. The table also shows the parameters used to monitor the effectiveness of sensitivity analyses introduced in Section 4.2.

The first thing to notice from Table 3 is that estimation and testing without imputation yield reasonably unbiased and appropriate type I error with either MCAR or MAR missing data. Standard errors for the bias estimates without imputation vary from 3.2 to 3.3 in Table 3.

When multiple imputation is used with δ=0 and either MCAR or MAR missing, we do see some bias, but do not see inflation of type I error. Standard errors for the bias estimates when δ=0 vary from 2.8 to 3.3 in Table 3. Once again, simulations with n=260 (reported in section 8.3 of the Supporting Information) support the notion that small sample bias in the imputation models is responsible for much if not all of the bias in the multiple imputation method with δ=0 and either MCAR or MAR missing. Note that with MNAR missing, any method in the table is not expected to exhibit appropriate estimation or testing. The last two scenarios confirm that MNAR missing can be quite problematic in that sense.

In evaluating Table 3 with regard to the sensitivity parameters defined in Section 4.2, notice that $p_{\text{SIR}}$ (rate of a *sensitivity interval of rejection*) is approximately controlled at 5% across all scenarios, including MNAR missing. Thus, a sensitivity analysis leading to a claim of significant treatment effect within any interval of δ is unlikely more than 5% of the time under the null (since Table 3 is a null treatment effect scenario). This is reassuring that the sensitivity analysis can provide a pullback from overly optimistic results obtained with missing even under some MNAR scenarios. We call this robust error control. Also note that $len_{\text{SIC}}$ (average half‐length of the sensitivity interval of concordance) is at least 0.429 across all of the scenarios. Thus, a sensitivity analysis will contain, on average, an interval of δ with half‐length at least 0.429 on which it agrees with the LMM model (3) without imputation. Note that $len_{\text{SIC}}$ is lowest in scenarios 3 and 4 with 20% or higher missing according to MAR. For most scenarios, the average $len_{\text{SIC}}$ is at least 0.74. This is reassuring that the sensitivity analysis often contains agreement over a wide range of δ with the un‐imputed analysis, even while providing the robust error control mentioned above.

Table 4 shows results of bias and power for testing via the LMM model (3) under a simulated data generation with treatment policy effect of $\beta_{A}^{*}=1000$. The same missingness scenarios indexed by k were used as in Table 3. The first thing to notice from Table 4 is that estimation and testing without imputation yield reasonably unbiased and appropriate power with MCAR or MAR missing data. In fact, power without imputation is at least 83% for all cases with MCAR or MAR missing. Standard errors for the bias estimates without imputation vary from 3.2 to 3.3 in Table 4.

**TABLE 4:** Estimated bias^a^ (% power, % different from no imputation^b^) for treatment policy effect (true effect=1000) from LMM model (3) with *SD‐sensitivity* version delta‐based sensitivity parameter δ applied to simulated SIU‐like trials ($n=130,\alpha^{*}=10\;000,\beta_{age}^{*}=-50,\beta_{0}^{*}=-4000,$ treatment policy effect $\beta_{A}^{*}=1000,\sigma_{c0}=\sigma_{c1}=50,\sigma_{e}=2000,M=10$). Missingness scenarios indexed by k and described in text.

Missing		^c^	^d^	^e^	^f^		δ		
Type	k	$p_{\text{miss}}$	$p_{\text{SIR}}$	$len_{\text{SIR}}$	$len_{\text{SIC}}$	No imp.	−1	0	1
MCAR	1	0.20	0.814	0.735	0.866	−5(85.1,−)	−17(73.8,13.6)	−18(85.1,1.0)	−22(73.8,13.5)
MCAR	2	0.40	0.791	0.692	0.823	−6(85.0,−)	−43(69.6,17.7)	−43(83.6,2.4)	−44(69.8,17.7)
MAR	3	0.21	0.623	0.301	0.323	−5(85.1,−)	−862(7.9,79.5)	−36(84.7,1.9)	789(99.7,14.6)
MAR	4	0.40	0.561	0.254	0.282	−5(83.5,−)	−937(6.4,80.2)	−92(80.5,4.9)	751 (99.7,16.2)
MAR	5	0.20	0.622	0.299	0.359	‐1 (83.9, —)	−994(9.4,81.1)	−143(81.1,5.5)	714 (96.2, 12.4)
MAR	6	0.21	0.610	0.280	0.330	5(83.4,−)	−1028(9.8,81.6)	−132(81.2,5.7)	765 (97.2,13.9)
MNAR	7	0.13	0.765	0.559	0.665	−59(87.1,−)	−499(37.9,50.3)	−80(83.0,4.5)	332 (82.7, 9.9)
MNAR	8	0.22	0.872	0.758	0.776	251 (97.5, —)	50(68.5,29.0)	−73(91.5,6.0)	−168(88.0,11.6)

^a^ Estimated over 10 000 replicated trials using (5); target rejection rate is 5% under null.

^b^ % of replications where the hypothesis test is different from the test without imputations.

^c^ $p_{\text{miss}}$ is average overall proportion of outcome values missing.

^d^ $p_{\text{SIR}}$ is rate of a sensitivity interval rejection.

^e^ $len_{\text{SIR}}$ is average half‐length of sensitivity interval of rejection.

^f^ $len_{\text{SIC}}$ is average half‐length of sensitivity interval of concordance.

When multiple imputation is used with δ=0 and either MCAR or MAR missing, we do see some bias. Standard errors for the bias estimates when δ=0 vary from 2.9 to 3.3 in Table 4. Once again, simulations with n=260 (reported in section 8.3 of the Supporting Information) support the notion that small sample bias in the imputation models is responsible for much if not all of the bias in the multiple imputation method with δ=0 and either MCAR or MAR missing. The last two scenarios confirm that MNAR missing can lead to bias with or without multiple imputation.

In evaluating Table 4 with regard to the sensitivity parameters defined in Section 4.2, notice that $p_{\text{SIR}}$ (rate of a *sensitivity interval of rejection*) is reasonably high across all scenarios. Thus, a sensitivity analysis leading to a claim of a significant treatment effect within some interval of δ is likely. This is reassuring that the sensitivity analysis can likely provide an interval of δ under which treatment benefit can be claimed if missingness is similar to any of the scenarios considered here. Note next that $len_{\text{SIC}}$ (average half‐length of *sensitivity interval of concordance*) is at least 0.282 across all scenarios. Thus, a sensitivity analysis will contain, on average, an interval of δ with half‐length at least 0.282 on which it agrees with the LMM model (3) without imputation. With MCAR missing, $len_{\text{SIC}}$ is above 0.8.

## 7 Discussion

We have demonstrated that *delta‐based* controlled imputation sensitivity analyses can be useful for investigating the robustness of a clinical trial's conclusions with regard to the missing values of the outcome of interest. In the setting of a repeatedly measured outcome variable, an LMM—for example, LMM model (3), can be used for primary analysis, and the sensitivity analysis can use imputation from the R package *mice* [12] with predictive mean matching. We also defined novel covariates (the last observed outcome before the given time) for the predictive models that are particularly useful in such settings. Finally, we introduced some parameters used to evaluate sensitivity analyses when investigating their properties via simulation. The concept of an SIR is useful to think about when investigating sensitivity analyses under null scenarios because it can be thought of as a robust incorrect rejection. Under alternative scenarios, the SIR can act as a type of robust power. An SIC can help see how often the testing conclusion might differ from the primary analysis (without imputation).

It is fairly well understood that linear mixed models in general require MAR missingness for valid statistical inference. However, some authors have used that fact to argue that multiple imputation is completely unnecessary [16]. We believe our results have definitively disproven that assertion. While one may always perform an analysis that relies on either MCAR or MAR missingness, it may not be wise to rely on the results with too much confidence. One of the best ways to increase your confidence in results in the presence of missing data is to perform a structured sensitivity analysis that includes MNAR missingness.

We focused on *delta‐based* controlled imputation, because in most cases it seems to make sense and is easily interpreted. We note, however, that *reference‐based* controlled imputation methods are also possible. These sensitivity methods specify a reference arm, whose predictive distribution is used in place of the actual arm when imputing for missing values. For *reference‐based* controlled imputation under the setup considered in this paper, specification of $f({\text{Y}}_{iM}|{\text{Y}}_{iO},{\text{R}}_{i},{\text{X}}_{i},\Omega )$ could be obtained by using the predictive distribution of ${\text{Y}}_{iM}^{*}$, the missing data considered as though it came from the reference arm (A=0).

Our treatment had a continuous outcome measured repeatedly. Controlled imputation methods with categorical outcomes are perhaps easier if *reference‐based*. One method [17] for repeatedly measured binary outcomes uses the logit from a logistic regression fit on the control participants' data for imputation. Controlled imputation using a *delta‐based* approach might be more difficult, but could add δ to the logit before making a random draw.

Our experience in devising sensitivity analyses for the SIU trial has led us to several key improvements in the analysis plan. Evaluating sensitivity analyses on simulated trial data has led us to modify our chosen method of imputation. Moreover, we have improved the characteristics of our sensitivity analysis by developing covariates that are useful in reducing bias from our imputed models. Finally, we have developed a better feeling for the level of robustness our primary trial result will have with regard to missing outcome data.

## Disclosure

The authors have nothing to report.

## Conflicts of Interest

The authors declare no conflicts of interest.

## Supporting information




**Data S1.** Supporting Information.

## Data Availability

The authors have nothing to report.
