# Supplementary material for: Sensitivity Analyses for Missing in Repeatedly Measured Outcome Data
Source: Stat Med. 2025 Oct 7;44(23-24):e70282. doi: 10.1002/sim.70282 (PMC12503996; doi:10.1002/sim.70282)
Supplement: Supplementary file 1 — Data S1. Supporting Information. [file SIM-44-0-s001.zip › Sensitivity_Analyses_for_Missing_Supp7.21.2025.pdf]

8 Supplement

8.1 Missingness Models for Simulation

The first stage data (weeks 1-12) generated from (6) was subjected to missingness by generating indicators of missing, R_{it}, according to the logistic model:

$$\begin{aligned}\log(\frac{p_{it}}{1-p_{it}}) = \alpha_m + \beta_{m0}(\frac{Y_{i,-1}+Y_{i0}}{2} - 6000) + \beta_{ma}(A_i - \frac{1}{2}) + \beta_{mt}(t-12) + \\ \beta_{ml}(Y_{il_t} - 10000) + \beta_{mla}(Y_{il_t} - 10000)(A_i - \frac{1}{2}) + \beta_{my}(Y_{it} - \mu_t) + \\ \beta_{mya}(Y_{it} - \mu_t)(A_i - \frac{1}{2}).\end{aligned} \tag{S1}$$

where p_{it} is the probability of missing, i.e. $R_{it} = 1$, μ_t is the average of $\{Y_{it} : i = 1, \dots, n\}$, and Y_{il_t} is the last previously observed value for participant i at time t. The second stage data (weeks 13-24) generated from (6) was subjected to missingness by generating indicators of missing, R_{it}, according to the logistic model:

$$\begin{aligned}\log(\frac{p_{it}}{1-p_{it}}) = \alpha_m + \beta_{m0}(\frac{Y_{i,-1}+Y_{i0}}{2} - 6000) + \beta_{ma}(A_i - \frac{1}{2}) + \beta_{mt}(t-12) + \\ \beta_{ml}(Y_{il_t} - 10000) + \beta_{mla}(Y_{il_t} - 10000)(A_i - \frac{1}{2}) + \beta_{my}(Y_{it} - \mu_t) + \\ \beta_{mya}(Y_{it} - \mu_t)(A_i - \frac{1}{2}) + \beta_{mr}(\frac{Y_{i21}+Y_{i22}+Y_{i23}+Y_{i24}}{4} - 10000).\end{aligned} \tag{S2}$$

Model (S1) generates data under an MNAR missingness mechanism that allows missingness in the first stage to depend on the potentially unobserved number of steps at the current week (through the β_{my} and β_{mya} terms). Model (S2) generates data under an MNAR missingness mechanism that allows missingness in the second stage to depend (in addition to the steps at the current week) on the potentially unobserved primary outcome (through the β_{mr} term). Models (S1)-(S2) allow a wide range of potential missingness mechanisms. Note that special cases of MCAR and MAR apply when certain parameters in (S1) and (S2) are zero. If all parameters in (S1) and (S2) other than α_m are zero, then missing is MCAR. If

$\beta_{my} = \beta_{mya} = \beta_{mr} = \beta_{m0} = 0$ in (S1) and (S2), then missing is MAR for the LMM (3). Note that with nonzero β_{ml} or β_{mla}, missing could be MAR for the LMM while being MNAR for the LM (1) since the LM does not contain the last observed value of Y before its outcome (Y_i, which is the average of $Y_{21}, Y_{22}, Y_{23}, Y_{24}$).

8.2 Using MICE for Multiple Imputation

We implement multiple imputation using the fully conditional specification with predictive mean matching, the default method in the R package **mice**. Broadly the algorithm is as follows: For each variable Y_{it} with missing data, let $\mathbf{W_{it}} = (AGE_i, Y_{i0}, A_i, Y_{i1}, \ldots, Y_{i,t-1}, Y_{i,t+1}, ..., Y_{i24}, Y_{il_t})$ be a vector of covariates for the imputation model to predict Y_{it}. Note that $\mathbf{W_{it}}$ includes the baseline covariates, treatment indicator, the Y_{ir} at other time-points r as well as the last observed Y_{it} (recall that Y_{il_t} is the last observed Y_{ir} for $r \leq t$). Let $\mathbf{Y_{obs,t}}$ be the subset of rows of $\mathbf{Y_{it}}$ for which Y_{it} is observed and $\mathbf{Y_{mis,t}}$ be the subset of rows of $\mathbf{Y_{it}}$ for which Y_{it} is missing. Similarly, let $\mathbf{W_{obs,t}}$ be the subset of rows of $\mathbf{W_{it}}$ for which Y_{it} is observed and $\mathbf{W_{mis,t}}$ be the subset of rows of $\mathbf{W_{it}}$ for which Y_{it} is missing.

1. As an initial step, replace each missing value with a random draw from that variable's empirical distribution. Retain the indicator of missing R_{it}.

2. Suppose there are R times, denoted $t_1, \ldots, t_R$, with at least one missing Y_{it}.

3. For $iter = 1, \ldots, 5$ do Steps 4-9.

4. For $j = 1, \ldots, R$ do Steps 5-9.

5. Fit the linear regression model of $\mathbf{W_{obs,t_j}}$ on $\mathbf{Y_{obs,t_j}}$. Let $\mathbf{\hat{Y}_{obs,t_j}} = \mathbf{\hat{\Gamma} W_{obs,t_j}}$ be the predicted values from the fitted model for the subjects with observed Y_{it_j} in the original dataset.

6. Make a random draw from the posterior predictive distribution of $\hat{\mathbf{\Gamma}}$ to produce a new set of coefficients $\dot{\mathbf{\Gamma}}$. This would be a random draw from a multivariate normal distribution with mean $\hat{\mathbf{\Gamma}}$ and the estimated covariance matrix of $\hat{\mathbf{\Gamma}}$ (with an additional random draw for the residual variance). This step is necessary to produce sufficient variability in the imputed values. For details, see Steps 1-8 of Algorithm 3.1 in Van Buuren (2018)[1].

7. Let $\hat{\mathbf{Y}}_{\mathbf{miss,t_j}} = \dot{\mathbf{\Gamma}}\mathbf{W}_{\mathbf{miss,t_j}}$ be the predicted values from the fitted model for the subjects with missing Y_{it_j} in the original dataset.

8. For each i with $R_{it_j} = 1$, find the five closest components of $\hat{\mathbf{Y}}_{\mathbf{obs,t_j}}$ to $(\hat{\mathbf{Y}}_{\mathbf{miss,t_j}})_i$. Draw randomly one of these five components, say the component corresponding to subject k. Impute the corresponding observed value Y_{kt_j}.

9. Update your dataset with the imputed values for Y_{it_j} .

8.3 Simulations with $n = 260$

In this section we present results of the MICE multiple imputation method with $\delta = 0$ and $n = 260$ and MAR missing over simulation scenarios of Tables 2-4.

Table 5 shows the results similar to Table 2.

Table 6 shows the results similar to Table 3.

Table 7 shows the results similar to Table 4.

8.4 A Worked Example of a Sensitivity Analysis

In this section we present a worked example from a simulated dataset that is analyzed using the sensitivity analysis evaluated by simulation in Section 4.

We simulated a dataset using the parameters of the second row of Table 4, where the intervention A policy effect is 1000 steps and there is 40% MCAR missing outcome values.

The data for the first participant is presented in Table 8. A summary of the dataset is presented in Table 9. In addition to $Y_1 - Y_{24}$ presented in Tables 8 & 9, we create $Y_{l_1} - Y_{l_{24}}$ the last observed Y_t before times $t = 1, \ldots, 24$. As examples, the first participant has $Y_{l_2} = Y_1 = 9487$, but $Y_{l_7} = Y_5 = 8650$.

We now follow the delta-based controlled imputation sensitivity analysis described in Section 4 with multiple imputation described in Section 8.2. We use $S = 8$ so that the combined grid of delta values is $\Delta = \{-1.0, -0.75, -0.5, -0.25, 0, 0.25, 0.5, 0.75, 1.0\}$. First we fit model (3) on the observed dataset. The results are shown in Table 10. Table 10 indicates that for example each year of age decreases average daily step counts by about 80. The estimated intervention A policy effect is the average of the estimated $\hat{\theta}_A = (\beta_{a21} + \ldots + \beta_{a24})/4 = 826.2$ with an estimated $\hat{sd}(\theta_A) = 317.2$ (not shown in the table). The test statistic for intervention policy effect is $\hat{\theta}_A / \hat{sd}(\theta_A) = 826.2/317.2 = 2.60$. This effect is statistically significant for a one-sided 2.5% test of positive treatment policy effect. The residual standard deviation from (3) is $\hat{SD} = 1335$. Thus $\delta_{max} = \lambda \cdot \hat{SD} = 2 \cdot 1335 = 2670$. For each $\delta \in \Delta$ we next use the General MI Algorithm from Section 6 with $M = 2$ to get $\hat{\theta}_A^{MI:\delta}$, its estimated variance $v\hat{a}r(\theta_A^{MI:\delta})$, and determine one-sided hypothesis tests for intervention benefit or harm (one-sided significance levels 0.025).

For $\delta = 0$ the first imputed dataset ($m = 1$) is summarized in Table 11. The second imputed dataset ($m = 2$) with $\delta = 0$ is summarized in Table 12. We next fit model (3) on the imputed datasets with $\delta = 0$. The results are shown in Tables 13 & 14. From Table 13 we get $\hat{\theta}_A^{1:\delta=0} = 854.7$ and from Table 14 we get $\hat{\theta}_A^{2:\delta=0} = 731.6$. The model estimated variance for $\hat{\theta}_A^{1:\delta=0}$ is $v\hat{a}r(\theta_A^{1:\delta=0}) = 92309.5$, while the model estimated variance for $\hat{\theta}_A^{2:\delta=0}$ is $v\hat{a}r(\theta_A^{2:\delta=0}) = 88866.4$. Therefore the multiple imputation estimate of intervention policy effect with $\delta = 0$ is $\hat{\theta}_A^{MI:\delta=0} = (854.7 + 731.6)/2 = 793.1$ and the model estimated variance is $v\hat{a}r(\theta_A^{model:\delta=0}) = (92309.5 + 88866.4)/2 = 90588.0$. We need to add the between imputation

variance, calculated as $BIV = 2(854.7 - 793.1)^2 = 7583.6$. We then apply Rubin's rules for a total variance of $v\hat{a}r(\theta_A^{MI:\delta=0}) = v\hat{a}r(\theta_A^{model:\delta=0}) + (3/2)BIV = 90588.0 + (1.5)7583.6 = 101963.4$. Taking square root gives $\hat{sd}(\theta_A^{MI:\delta=0}) = \sqrt{101963.4} = 319.3$. The test statistic for the sensitivity analysis with $\delta = 0$ is $\hat{\theta}_A^{MI:\delta=0}/\hat{sd}(\theta_A^{MI:\delta=0}) = 793.1/319.3 = 2.48$. Thus, like the one-sided test based on the observed dataset, the $\delta = 0$ sensitivity analysis rejects the null hypothesis in favor of a positive intervention policy effect.

We continue as described above for $\delta \in \Delta$. We describe results for the only δ for which we do not reject the one-sided hypothesis test for positive treatment policy effect. For $\delta = -1$ the first imputed dataset ($m = 1$) is summarized in Table 15. The second imputed dataset ($m = 2$) with $\delta = -1$ is summarized in Table 16. Note that we start from the same two datasets obtained with $\delta = 0$ instead of generating fresh imputed datasets (to reduce computational burden). Thus, the first imputed dataset for $\delta = -1$ is exactly the first imputed dataset for $\delta = 0$ with $\delta_{max} = 2670$ subtracted from each Y_{it} that was missing in the observed dataset. We next fit model (3) on the imputed datasets with $\delta = -1$. The results are shown in Tables 17 & 18. From Table 17 we get $\hat{\theta}_A^{1:\delta=-1} = 684.9$ and from Table 18 we get $\hat{\theta}_A^{2:\delta=-1} = 588.2$. The model estimated variance for $\hat{\theta}_A^{1:\delta=-1}$ is $v\hat{a}r(\theta_A^{1:\delta=-1}) = 124425.7$, while the model estimated variance for $\hat{\theta}_A^{2:\delta=-1}$ is $v\hat{a}r(\theta_A^{2:\delta=-1}) = 120124.6$. Therefore the multiple imputation estimate of intervention policy effect with $\delta = -1$ is $\hat{\theta}_A^{MI:\delta=-1} = (684.9+588.2)/2 = 636.5$ and the model estimated variance is $v\hat{a}r(\theta_A^{model:\delta=-1}) = (124425.7+120124.6)/2 = 122275.2$. We need to add the between imputation variance, calculated as $BIV = 2(684.9 - 636.5)^2 = 4670.4$. We then apply Rubin's rules for a total variance of $v\hat{a}r(\theta_A^{MI:\delta=-1}) = v\hat{a}r(\theta_A^{model:\delta=-1}) + (3/2)BIV = 122275.2 + (1.5)4670.4 = 129280.8$. Taking square root gives $\hat{sd}(\theta_A^{MI:\delta=-1}) = \sqrt{129280.8} = 359.6$. The test statistic for the sensitivity analysis with $\delta = -1$ is $\hat{\theta}_A^{MI:\delta=-1}/\hat{sd}(\theta_A^{MI:\delta=-1}) = 636.5/359.6 = 1.77$. Thus, unlike the one-sided test based on the observed dataset, the $\delta = -1$ sensitivity analysis fails to reject

the null hypothesis in favor of a positive intervention policy effect.

Since this was simulated data and the absolute maximal δ leading to rejection for all smaller δ (as well as the observed data) was 0.75, there was an *sensitivity interval of rejection* of half-length 0.75. Similarly, there was a *sensitivity interval of concordance* of half-length 0.75.

If evaluating the sensitivity analysis on real data, we would make the following conclusions. The linear mixed model (3) shows a significant positive intervention policy effect, which is valid for MAR missingness. The sensitivity analysis shows that the result is robust to MNAR missingness according to a pattern mixture where the unobserved values all deviated in either direction from the MAR expected value by up to 0.75 $\delta_{max} = 2003$ steps/day.

SUPPLEMENT REFERENCES

1. van Buuren S. Flexible Imputation of Missing Data. 2nd ed. CRC Press. Boca Raton FL: Chapman & Hall, 2018.

Table 5: Estimated bias±2SE[♯] for treatment policy effect from LMM model (3) with the MICE imputation method applied to simulated SIU-like trials ($n = 260, \alpha^* = 10000, \beta^*_{age} = -50, \beta^*_0 = -4000, \beta^*_A = 0, \sigma_{c0} = \sigma_{c1} = 50, \sigma_e = 2000, M = 1$). Missingness for MAR scenarios from Supplement models (S1) and (S2) with zero parameters except those listed in table and $\beta_{ma} = 1, \beta_{mt} = 1/12$.

						Imputation Method
Missing	α_m	β_{ml}	β_{mla}	$p_{miss}{}^{\perp}$	No imp.	MICE$^{\nabla}$
MAR	-1.5	0	0	0.21	0 ± 4.7	-1 ± 4.7
MAR	-0.5	0	0	0.40	-1 ± 4.8	0 ± 4.7
MAR	-1.5	0.001	0	0.15	1 ± 4.8	1 ± 4.5
MAR	-1.5	0.001	0.001	0.15	4 ± 4.8	10 ± 4.5

♯ Estimated over 10000 replicated trials using (5)
$^{\perp}$ p_{miss} is average overall proportion of outcome values missing
$^{\nabla}$ Imputation from **mice** R package with predictive mean matching described in Section 8.1

Table 6: Estimated bias[♯] (% type I error[†], % different from no imputation[⋄]) for treatment policy effect from LMM model (3) with sensitivity parameter $\delta = 0$ applied to simulated SIU-like trials ($n = 260, \alpha^* = 10000, \beta^*_{age} = -50, \beta^*_0 = -4000, \beta^*_A = 0, \sigma_{c0} = \sigma_{c1} = 50, \sigma_e = 2000, M = 10$). Missingness scenarios indexed by k and described in text of paper.

Missing Type	k	$^\perp$ p_{miss}	No imp.	δ 0
MAR	3	0.21	0(5.4,-)	0(5.4,0.7)
MAR	4	0.40	-1(5.4,-)	-1(5.0,1.5)
MAR	5	0.15	1(5.4,-)	1(5.4,1.6)
MAR	6	0.15	4(5.3,-)	10(5.2,1.4)

♯ Estimated over 10000 replicated trials using (5); target rejection rate is 5% under null
† Standard error of type I error estimates over the 10000 replications is 0.22%
⋄ % of replications where hypothesis test different than test without imputations
$^\perp$ p_{miss} is average overall proportion of outcome values missing

Table 7: Estimated bias[♯] (% power, % different from no imputation[⋄]) for treatment policy effect from LMM model (3) with sensitivity parameter $\delta = 0$ applied to simulated SIU-like trials ($n = 260, \alpha^* = 10000, \beta^*_{age} = -50, \beta^*_0 = -4000, \beta^*_A = 1000, \sigma_{c0} = \sigma_{c1} = 50, \sigma_e = 2000, M = 10$). Missingness scenarios indexed by k and described in text of paper.

Missing Type	k	$^\perp$ p_{miss}	No imp.	δ 0
MAR	3	0.21	0(98.8,-)	-15(98.9,0.2)
MAR	4	0.40	-1(98.7,-)	-42(98.5,0.6)
MAR	5	0.20	3(98.7,-)	-84(98.6,0.5)
MAR	6	0.21	6(98.5,-)	-47(99.0,0.6)

♯ Estimated over 10000 replicated trials using (5); target rejection rate is 5% under null
⋄ % of replications where hypothesis test different than test without imputations
$^\perp$ p_{miss} is average overall proportion of outcome values missing

Table 8: Simulated first participant with treatment policy effect (true effect=1000) from LMM model (6) with 40% MCAR missing ($n = 130, \alpha^* = 10000, \beta^*_{age} = -50, \beta^*_0 = -4000, \beta^*_A = 1000, \sigma_{c0} = \sigma_{c1} = 50, \sigma_e = 2000$).

BA	A	Y_0	Y_1	Y_2	Y_3	Y_4	Y_5	Y_6	Y_7	Y_8	Y_9	Y_{10}	Y_{11}	Y_{12}
26	0	5694	9487	8966	9090	9073	8650	NA	8786	NA	NA	NA	NA	NA

Y_{13}	Y_{14}	Y_{15}	Y_{16}	Y_{17}	Y_{18}	Y_{19}	Y_{20}	Y_{21}	Y_{22}	Y_{23}	Y_{24}
8714	8475	7904	7988	NA	NA	NA	NA	6483	6760	6679	6368

Table 9: Simulated dataset (mean, median, # NA) with treatment policy effect (true effect=1000) from LMM model (6) with 40% MCAR missing ($n = 130, \alpha^* = 10000, \beta^*_{age} = -50, \beta^*_0 = -4000, \beta^*_A = 1000, \sigma_{c0} = \sigma_{c1} = 50, \sigma_e = 2000$).

BA	A	Y_0	Y_1	Y_2	Y_3	Y_4	Y_5
29.5,28.5,0	0.47,0,0	4654,4744,0	9045,9174,45	9215,9200,50	9053,9090,45	9210,9229,52	9049,8982,49

Y_6	Y_7	Y_8	Y_9	Y_{10}	Y_{11}	Y_{12}	Y_{13}
9218,9225,63	9032,8905,49	9297,9284,64	9404,9324,49	9154,8966,53	9161,9063,48	9135,9066,48	9378,9174,45

Y_{14}	Y_{15}	Y_{16}	Y_{17}	Y_{18}	Y_{19}	Y_{20}	Y_{21}
9064,9121,50	9308,9360,47	9174,9240,44	9343,9389,60	8976,8788,48	9028,8984,52	8971,8730,58	9202,9322,60

Y_{22}	Y_{23}	Y_{24}
9033,8895,46	9049,8928,45	8919,8638,60

Table 10: Estimated coefficients from fit of LMM (3) on observed dataset (see Table 9).

$\hat{\beta}_{age}$	$\hat{\beta}_0$	$\hat{\beta}_{a1}$	$\hat{\beta}_{a2}$	$\hat{\beta}_{a3}$	$\hat{\beta}_{a4}$	$\hat{\beta}_{a5}$	$\hat{\beta}_{a6}$	$\hat{\beta}_{a7}$	$\hat{\beta}_{a8}$	$\hat{\beta}_{a9}$	$\hat{\beta}_{a10}$	$\hat{\beta}_{a11}$	$\hat{\beta}_{a12}$	$\hat{\beta}_{a13}$
-79.7	7016	939	973	962	1061	1061	1141	1059	961	1078	1052	1142	1104	1026

$\hat{\beta}_{a14}$	$\hat{\beta}_{a15}$	$\hat{\beta}_{a16}$	$\hat{\beta}_{a17}$	$\hat{\beta}_{a18}$	$\hat{\beta}_{a19}$	$\hat{\beta}_{a20}$	$\hat{\beta}_{a21}$	$\hat{\beta}_{a22}$	$\hat{\beta}_{a23}$	$\hat{\beta}_{a24}$	$\hat{\beta}_1$	$\hat{\beta}_t$	$\hat{\beta}_{t2}$	$\hat{\beta}_{t3}$
1121	1049	989	951	803	840	962	868	774	847	817	11027	80.4	-47.1	2.2

Table 11: First imputed dataset (mean, median, # NA) from MICE with $\delta = 0$.

BA	A	Y_0	Y_1	Y_2	Y_3	Y_4	Y_5
29.5,28.5,0	0.47,0,0	4654,4744,0	9116,9235,0	9203,9455,0	9129,9224,0	9203,9170,0	9194,9278,0

Y_6	Y_7	Y_8	Y_9	Y_{10}	Y_{11}	Y_{12}	Y_{13}
9246,9225,0	9174,9032,0	9200,9118,0	9275,9281,0	9170,8996,0	9205,9119,0	9284,9242,0	9147,9034,0

Y_{14}	Y_{15}	Y_{16}	Y_{17}	Y_{18}	Y_{19}	Y_{20}	Y_{21}
9180,9064,0	9304,9345,0	9212,9310,0	9105,9046,0	9036,8957,0	9120,9189,0	9131,8920,0	9148,9091,0

Y_{22}	Y_{23}	Y_{24}
9042,8976,0	9030,8774,0	9044,8920,0

Table 12: Second imputed dataset (mean, median, # NA) from MICE with $\delta = 0$.

BA	A	Y_0	Y_1	Y_2	Y_3	Y_4	Y_5
29.5,28.5,0	0.47,0,0	4654,4744,0	9125,9235,0	9192,9192,0	9143,9279,0	9216,9216,0	9217,9201,0

Y_6	Y_7	Y_8	Y_9	Y_{10}	Y_{11}	Y_{12}	Y_{13}
9233,9240,0	9081,8876,0	9071,9118,0	9247,9282,0	9269,9084,0	9179,9067,0	9271,9228,0	9245,9131,0

Y_{14}	Y_{15}	Y_{16}	Y_{17}	Y_{18}	Y_{19}	Y_{20}	Y_{21}
9223,9298,0	9270,9345,0	9211,9282,0	9115,9150,0	9023,8781,0	9134,9002,0	9141,8926,0	9085,9055,0

Y_{22}	Y_{23}	Y_{24}
9057,8973,0	9014,8837,0	9053,8861,0

Table 13: Estimated coefficients from fit of LMM (3) on first imputed dataset (see Table 11).

$\hat{\beta}_{age}$	$\hat{\beta}_0$	$\hat{\beta}_{a1}$	$\hat{\beta}_{a2}$	$\hat{\beta}_{a3}$	$\hat{\beta}_{a4}$	$\hat{\beta}_{a5}$	$\hat{\beta}_{a6}$	$\hat{\beta}_{a7}$	$\hat{\beta}_{a8}$	$\hat{\beta}_{a9}$	$\hat{\beta}_{a10}$	$\hat{\beta}_{a11}$	$\hat{\beta}_{a12}$	$\hat{\beta}_{a13}$
-65.9	6611	892	953	926	1029	1058	1195	987	944	1052	940	1100	1129	967

$\hat{\beta}_{a14}$	$\hat{\beta}_{a15}$	$\hat{\beta}_{a16}$	$\hat{\beta}_{a17}$	$\hat{\beta}_{a18}$	$\hat{\beta}_{a19}$	$\hat{\beta}_{a20}$	$\hat{\beta}_{a21}$	$\hat{\beta}_{a22}$	$\hat{\beta}_{a23}$	$\hat{\beta}_{a24}$	$\hat{\beta}_1$	$\hat{\beta}_t$	$\hat{\beta}_{t2}$	$\hat{\beta}_{t3}$
1036	1080	950	825	786	804	821	926	791	857	845	10647	20.1	56.9	-40.3

Table 14: Estimated coefficients from fit of LMM (3) on second imputed dataset (see Table 12).

$\hat{\beta}_{age}$	$\hat{\beta}_0$	$\hat{\beta}_{a1}$	$\hat{\beta}_{a2}$	$\hat{\beta}_{a3}$	$\hat{\beta}_{a4}$	$\hat{\beta}_{a5}$	$\hat{\beta}_{a6}$	$\hat{\beta}_{a7}$	$\hat{\beta}_{a8}$	$\hat{\beta}_{a9}$	$\hat{\beta}_{a10}$	$\hat{\beta}_{a11}$	$\hat{\beta}_{a12}$	$\hat{\beta}_{a13}$
-67.8	6664	964	984	890	1073	1037	1093	865	800	965	1094	1071	1104	1026

$\hat{\beta}_{a14}$	$\hat{\beta}_{a15}$	$\hat{\beta}_{a16}$	$\hat{\beta}_{a17}$	$\hat{\beta}_{a18}$	$\hat{\beta}_{a19}$	$\hat{\beta}_{a20}$	$\hat{\beta}_{a21}$	$\hat{\beta}_{a22}$	$\hat{\beta}_{a23}$	$\hat{\beta}_{a24}$	$\hat{\beta}_1$	$\hat{\beta}_t$	$\hat{\beta}_{t2}$	$\hat{\beta}_{t3}$
1088	1076	961	894	686	828	853	842	725	736	624	10654	296.3	-306.9	91.4

Table 15: First imputed dataset (mean, median, # NA) from MICE with $\delta = -1$.

BA	A	Y_0	Y_1	Y_2	Y_3	Y_4	Y_5
29.5,28.5,0	0.47,0,0	4654,4744,0	8192,8177,0	8176,7996,0	8204,8355,0	8135,7927,0	8187,8122,0

Y_6	Y_7	Y_8	Y_9	Y_{10}	Y_{11}	Y_{12}	Y_{13}
7952,7866,0	8168,8146,0	7886,8240,0	8269,8106,0	8081,7934,0	8220,8133,0	8298,8237,0	8223,8456,0

Y_{14}	Y_{15}	Y_{16}	Y_{17}	Y_{18}	Y_{19}	Y_{20}	Y_{21}
8153,7828,0	8338,8228,0	8308,8276,0	7873,7742,0	8050,8364,0	8052,8388,0	7940,7950,0	7916,7908,0

Y_{22}	Y_{23}	Y_{24}
8097,7843,0	8105,8296,0	7812,7821,0

Table 16: Second imputed dataset (mean, median, # NA) from MICE with $\delta = -1$.

BA	A	Y_0	Y_1	Y_2	Y_3	Y_4	Y_5
29.5,28.5,0	0.47,0,0	4654,4744,0	8201,8177,0	8165,7996,0	8219,8386,0	8148,8137,0	8211,8110,0

Y_6	Y_7	Y_8	Y_9	Y_{10}	Y_{11}	Y_{12}	Y_{13}
7939,7842,0	8075,8148,0	7756,7736,0	8240,8136,0	8180,8094,0	8193,8322,0	8285,8272,0	8321,8542,0

Y_{14}	Y_{15}	Y_{16}	Y_{17}	Y_{18}	Y_{19}	Y_{20}	Y_{21}
8196,7922,0	8305,8244,0	8308,8252,0	7883,7745,0	8037,8115,0	8066,8414,0	7950,7874,0	7852,7896,0

Y_{22}	Y_{23}	Y_{24}
8112,7898,0	8089,8246,0	7821,7807,0

Table 17: Estimated coefficients from fit of LMM (3) on first imputed dataset (see Table 15).

$\hat{\beta}_{age}$	$\hat{\beta}_0$	$\hat{\beta}_{a1}$	$\hat{\beta}_{a2}$	$\hat{\beta}_{a3}$	$\hat{\beta}_{a4}$	$\hat{\beta}_{a5}$	$\hat{\beta}_{a6}$	$\hat{\beta}_{a7}$	$\hat{\beta}_{a8}$	$\hat{\beta}_{a9}$	$\hat{\beta}_{a10}$	$\hat{\beta}_{a11}$	$\hat{\beta}_{a12}$	$\hat{\beta}_{a13}$
-60.8	6460	627	670	791	726	971	963	909	487	955	802	1400	1249	1342

$\hat{\beta}_{a14}$	$\hat{\beta}_{a15}$	$\hat{\beta}_{a16}$	$\hat{\beta}_{a17}$	$\hat{\beta}_{a18}$	$\hat{\beta}_{a19}$	$\hat{\beta}_{a20}$	$\hat{\beta}_{a21}$	$\hat{\beta}_{a22}$	$\hat{\beta}_{a23}$	$\hat{\beta}_{a24}$	$\hat{\beta}_1$	$\hat{\beta}_t$	$\hat{\beta}_{t2}$	$\hat{\beta}_{t3}$
1226	1172	1163	590	718	817	350	412	853	887	587	9799	-1051.8	1006.0	-289.9

Table 18: Estimated coefficients from fit of LMM (3) on second imputed dataset (see Table 16).

$\hat{\beta}_{age}$	$\hat{\beta}_0$	$\hat{\beta}_{a1}$	$\hat{\beta}_{a2}$	$\hat{\beta}_{a3}$	$\hat{\beta}_{a4}$	$\hat{\beta}_{a5}$	$\hat{\beta}_{a6}$	$\hat{\beta}_{a7}$	$\hat{\beta}_{a8}$	$\hat{\beta}_{a9}$	$\hat{\beta}_{a10}$	$\hat{\beta}_{a11}$	$\hat{\beta}_{a12}$	$\hat{\beta}_{a13}$
-63.5	6540	699	707	766	783	963	874	799	354	876	961	1372	1222	1397

$\hat{\beta}_{a14}$	$\hat{\beta}_{a15}$	$\hat{\beta}_{a16}$	$\hat{\beta}_{a17}$	$\hat{\beta}_{a18}$	$\hat{\beta}_{a19}$	$\hat{\beta}_{a20}$	$\hat{\beta}_{a21}$	$\hat{\beta}_{a22}$	$\hat{\beta}_{a23}$	$\hat{\beta}_{a24}$	$\hat{\beta}_1$	$\hat{\beta}_t$	$\hat{\beta}_{t2}$	$\hat{\beta}_{t3}$
1271	1160	1165	651	611	838	384	338	805	797	412	9843	-890.1	812.5	-221.5
